# Gender differences in mental distress and affect balance during the first wave of COVID-19 pandemic in Spain

**DOI:** 10.1007/s12144-022-03282-w

**Published:** 2022-06-04

**Authors:** M. Pilar Matud, Jesús Zueco, Amelia Díaz, Mª José del Pino, Demelsa Fortes

**Affiliations:** 1 Department of Clinical Psychology, Psychobiology and Methodology, Facultad de Psicología y Logopedia, Universidad de La Laguna, Apartado 456, 38200 La Laguna, Spain; 2 Department of Microbiology, University of Valencia, Valencia, Spain; 3 Department of Personality, Evaluation and Psychological Treatments, University of Valencia, Valencia, Spain; 4 Department of Sociology, University Pablo de Olavide, Seville, Spain; 5 European University of the Canary Islands, Santa Cruz de Tenerife, Spain

**Keywords:** COVID-19, Gender, Mental health, Subjective well-being, Self-esteem, Social support

## Abstract

The COVID-19 pandemic is a major threat to the health and well-being of people around the world that has impacted freedom of movement, social interaction and the economy. The aim of the present work was twofold: first, to study the presence of mental distress, positive and negative experiences and affect balance in women and men in Spain in two different phases of the COVID-19 pandemic, the initial “first state of alarm” phase, characterized by maximum restrictions, and in the “new normal” phase with minimal restrictions, and second, to study the protective role of age, educational level, self-esteem, marital status and social support against mental distress, and as factors that increase the affect balance of women and men in the above mentioned phases of the first wave of the COVID- 19 pandemic in Spain. The study sample consisted of 652 women and 652 men from the general population, aged between 18 and 88 years, who were evaluated through self-reports. Results show greater mental distress in women than men but, strikingly, the magnitude of such differences were greater in the “new normal” phase than in the maximum restriction phase. In addition, in this last phase, women also experienced more negative feelings and less affect balance than men. High self-esteem and social support were also found to be protective factors for mental health, both in women and men, during the two phases of the pandemic studied. In conclusion, our study shows that the COVID-19 pandemic has especially impacted the well-being of women.

## Introduction

The first human cases of coronavirus disease (COVID-19), an infectious disease caused by a novel coronavirus named SARS-CoV-2, were reported in December 2019 in Wuhan City, China (World Health Organization, WHO, 2020) with the virus spreading rapidly around the world. The 2019-CoV-2 outbreak led to the implementation of extraordinary public health measures to reduce the spread of the virus within China and elsewhere, although there was heterogeneity in the public health measures implemented across countries.

### State of alarm and new normality COVID-19 phases in Spain

After the first COVID-19 case was diagnosed on January 31st 2020, the disease spread rapidly in Spain, which experienced one of the worst first waves of the COVID-19 in Europe and the world (Working group for the surveillance and control of COVID-19 in Spain, 2020). In an attempt to control the situation, a national lockdown was implemented on March 15th 2020, based on the State of Alarm Royal Decree 463/2020 of March 14th, which was extended until 00:00 h of June 21, 2020 (Official State Gazette, 2020). The state of alarm implied a series of very restrictive measures that included, among others, the limitation of the freedom of movement of people; the suspension of non-essential commercial activity and the suspension of face-to-face educational activity in all stages of education.

Although lockdown restrictions were eased from April 26th and, as of May 11th, the restrictive measures began to slowly relax with the application of the different phases of the Transition Plan to a “new normal” situation, the restrictive measures had profoundly affected every aspect of day-to-day life. The “new normal” situation was in force in Spain from June 21st 2020 to October 25th 2020, the date on which the Spanish government again declared a state of alarm throughout the national territory to contain the spread of new infections. During the “new normal” situation in Spain, all activities were allowed as long as an interpersonal distance of at least 1.5 m could be kept or, if this social distance could not be maintained, “adequate hygiene measures” were implemented. In addition, other measures were imposed, such as the mandatory use of facemasks for all people over six years of age on public spaces, both outdoor and indoor.

### COVID-19 pandemic and mental health

The COVID-19 pandemic is a global challenge that has had a significant impact not only on physical health but has also significantly altered people’s lives and affected multiple aspects of the global, public and private economy (Xiong et al., 2020). The uncertainties and fears associated with the virus outbreak, along with public health measures to contain the pandemic, have produced a significant increase in mental health issues (Lambert et al., 2020). Studies performed in different countries during the pandemic have highlighted a wide range of psychological problems in the general population, affecting not only health workers and COVID-19 patients but also patients suffering chronic diseases, older adults and population submitted to quarantine (de Sousa et al., 2021; Xiong et al., 2020; Wu et al., 2021). However, a high degree of heterogeneity was found in review studies and meta-analysis, with prevalence varying depending on the health problem analyzed and the population studied (de Souza et al., 2021; Wu et al., 2021).

According to a recently published meta-review of 18 prevalence meta-analyses evaluating the impact of COVID-19 pandemic, the prevalence of mental health problems ranged from 20 to 36% and the global prevalence of psychological distress was 28.25% (de Souza et al., 2021). The prevalence data found in this meta-review were lower than those found by Wu et al. (2021) in a meta-analysis of 66 studies with a total of 221,970 participants where the overall pooled prevalence of distress was 41.1%. Mental health problems were higher in health care workers, noninfectious chronic disease patients, COVID-19 patients, and quarantined persons (Wu et al., 2021). Other risk factors for mental problems found in various studies were gender (female) (Gibson et al., 2021; Kolakowsky-Hayner et al., 2021; Oryan et al., 2021; Pierce et al., 2020; Rens et al., 2021; Salfi et al., 2020), age (younger people) (Gibson et al., 2021; Huang & Zhao, 2020; Oryan et al., 2021; Pierce et al., 2020) and low social support (Oryan et al., 2021; Rens et al., 2021).

The COVID-19 pandemic has not only increased the risk of the general population to develop psychopathology and distress, but it is also a threat to their mental well-being (Choi et al., 2021; Khan et al., 2021; Wanberg et al., 2020). Subjective well-being reflecting an overall evaluation of the quality of a person’s life from that person’s own perspective, referring to the extent to which a person believes or feels that her/his life is going well (Diener et al., 2018). Subjective well-being has two major components: life satisfaction, which is the cognitive component, and experienced emotions, which is the affective component and depends primarily on the frequency of positive and negative affective experiences (Diener et al., 1991). Positive and negative feelings scores can be combined to create an affect balance score, which often has been represented as a difference score (positive minus negative affect) (Diener et al., 2010). Affect balance score is positively associated with life satisfaction (du Plessis & Guse, 2016) and predicts daily emotional experience (Veilleux et al., 2020). In addition to being important in itself, a number of reviews and meta-analyzes have shown that high subjective well-being benefits health, longevity, citizenship, and social relationships (Diener, 2012; Diener & Chan, 2011). Furthermore, the evidence suggests that “positive affect—the hallmark of well-being—may be the cause of many of the desirable characteristics, resources, and successes correlated with happiness” (Lyubomirsky et al., 2005, p. 803).

Research carried out before the COVID-19 pandemic has shown that mental distress and positive experience are influenced by personal and social factors, including self-esteem, social support and educational level (Byles et al., 2012; Matud, 2019; Matud et al., 2015; Orth et al., 2016). There is evidence that self-esteem influences success and well-being in several areas, including work, relationships and physical and mental health (Orth, 2017; Orth et al., 2016; von Soest et al., 2018). In research carried out during the COVID-19 pandemic, it has been found that self-esteem plays an important role in adaptation to the environment (Zhao et al., 2021), moderating and/or mediating the relationship between the threat perceived by the pandemic and the adverse psychological consequences (Lin & Chen, 2021; Rossi et al., 2020; Zhao et al., 2021). Additionally, social support is associated not only with mental health (Harandi et al., 2017) but also with morbidity and mortality (Holt-Lunstad et al., 2010). Furthermore, social support has also been associated with subjective well-being (Siedlecki et al., 2014). Studies conducted during the COVID-19 pandemic have also found that high social support is a protective factor for mental health (Grey et al., 2020; Li et al., 2021; Oryan et al., 2021; Szkody et al., 2021).

The role of level of education is more controversial, although some studies have found an association between a lower level of education and greater psychological distress (Byles et al., 2012; Talala et al., 2008), others have not found such an association (Kosidou et al., 2011; Molarius & Granstrom, 2018). Studies conducted during the COVID-19 pandemic have also shown non-conclusive association between education and psychological distress (see review by Wang et al., 2020). A study conducted in the general population of Pakistan found that education is not associated with well-being (Khan et al., 2021), and Wanberg et al., (2020) found that people with higher education experienced a greater increase in their depressive symptomatology and a greater decrease in their satisfaction with life than those with a lower level of education, when the before and during pandemic phases were compared.

### The present study

Although there are many studies on COVID-19 and its impact on people’s physical and mental health, few studies have incorporated a gender-based analysis. Besides, policies and public health efforts haven’t addressed the gender impact of the COVID-19 outbreak (Wenham et al., 2020). In addition, most of the studies have focused on the evaluation of the presence of psychopathology, psychological problems and distress but have not directly evaluated the presence of possible desirable experiences. Another important issue of the COVID-19 pandemic is the wide variation in the incidence and prevalence of the disease observed over time in each country and between countries, that has prompted governments to adjust the restrictions imposed to the population to the specific circumstances of the moment, with periods of time (phases) characterized by rigorous restrictions in the activities that are allowed and others where there is a certain “normality” with minimal restrictions.

Therefore, the present study focuses on the analysis, following a gender perspective, of the presence of mental distress, positive and negative experiences and affect balance in women and men in Spain in two different phases of the first wave of the COVID-19 pandemic; namely, the first state of alarm, a phase with severe restrictions, and what was referred to as “the new normal”, a phase characterized by minimal restrictions. In addition, the self-esteem and social support of women and men in both phases were also analyzed. A second aim of our study was to determine the protective role of age, educational level, marital status, self-esteem and social support against mental distress and as factors that increase the affect balance of women and men in the two above mentioned phases of the first wave of the COVID-19 pandemic in Spain.

## Method

### Participants

The participants consisted of 652 women and 652 men from the Spanish general population, ranging in age from 18 to 88 years old (*M* = 39.10 years, *SD* = 13.95 years). Half of them (326 women and 326 men) completed the questionnaires during the state of alarm phase and the other half (326 women and 326 men) during the “new normal” phase. Table 1 shows the main sociodemographic characteristics by gender for each phase. As can be observed, women and men did not differ in age, education, marital status and occupation in either phase. Although there was diversity in sociodemographic, almost a half of the participants had university degrees, which occurred in 48.0% of women and 46.0% of men. Just over half of the sample (52.9% of the women and 53.9% of the men) were married or living with their partner, slightly more than a third was never married and the rest were separated, divorced or widowed. The data related to the occupation showed that the most of the participants (75.6%), both in women and men, were employed, although 16.3% of the sample were unemployed, and 4.6% of women and 5.5% of men were retired.

**Table 1:** Sociodemographic characteristics of the women and men groups in state of alarm and in “new normal” phases

Measure	State of alarm phase						“New normal” phase					
	Women (*n* = 326)		Men (*n* = 326)				Women (*n* = 326)		Men (*n* = 326)			
	*n*	*%*	*n*	*%*	Χ^2^	*p*	*n*	*%*	*n*	*%*	Χ^2^	*p*
Education:												
Elementary studies or without studies	31	9.5	22	6.8	3.41	0.333	21	6.4	19	5.8	1.36	0.715
Secondary studies	29	8.9	27	8.3			31	9.5	40	12.3		
High school/professional training	107	32.8	126	38.8			120	36.8	117	36.0		
University degree	159	48.8	150	46.2			154	47.2	149	45.8		
Non data			1						1			
Marital status:												
Never married	118	36.2	125	38.3	2.99	0.224	119	36.5	125	38.5	1.01	0.605
Married/partnered	170	52.1	176	54.0			175	53.7	175	53.8		
Separated/divorced/widowed	38	11.7	25	7.7			32	9.8	25	7.7		
Non data									1			
Occupation:												
Working	250	76.7	248	76.1	0.13	0.989	243	74.5	247	75.8	3.20	0.362
Unemployed	53	16.3	53	16.3			53	16.3	53	16.3		
Retired	16	4.9	18	5.5			14	4.3	18	5.5		
Other	7	2.1	7	2.1			16	4.9	8	2.5		
	*M*	*SD*	*M*	*SD*	*t*	*p*	*M*	*SD*	*M*	*SD*	*t*	*p*
Age	39.37	13.59	38.36	14.57	0.91	0.363	39.56	13.33	39.13	14.30	0.39	0.694
Number of children	0.95	1.14	0.72	1.07	2.65	0.008	0.79	0.99	0.84	1.21	-0.61	0.540

### Procedure

All participants were volunteers, and were not remunerated for their participation. Convenience and snowball samplings were used. Participants were recruited through the social net of the researches and that of Psychology and Sociology degree university students who received course credits for that task. Data were collected through an online survey utilizing a Google Form between June 1st and June 20th, 2020, when the state of alarm was still in force (Time 1, state of alarm) and between October 15th and October 25th, 2020, corresponding to the “new normal” phase in Spain (Time 2, “new normal”), some four months separating the two tests. Participants were sent a link electronically to complete the study online from their personal computer or the WhatsApp application at their convenience. The data collection was carried out identically in the two phases but the link to complete the questionnaire was sent only once to each participant, so that participants in Time 1 are different from those in Time 2, although their sociodemographic characteristics were controlled to be similar. The mean age of the people who participated in Time 1 was 38.87 years (*SD* = 14.09) and those in Time 2 was 39.34 (*SD* = 13.81), *t*(1300) = -0.62, p =. 54; and the number of children of the people who participated in Time 1 was 0.83 (*SD* = 1.11) and Time 2 was 0.81 (*SD* = 1.10), *t*(1266) = 0.32, *p* =. 75. People in both phases showed also not significantly different in education, χ^2^(3, *N* = 1302) = 3.68, *p* = .30; marital status χ^2^(2, *N* = 1303) = 0.32, *p* = .85; nor in occupation χ^*2*^(3, *N* = 1304) = 2.76, *p* = .43.

The sample used in this study was randomly selected from a larger one on the psychological impact of the COVID-19 pandemic in Spain using the following criteria (in addition to the test having being carried out in the cited time phases): (1) That the participants were 18 years of age or older but not students. (2) That women and men did not differ statistically significantly in age, educational level, occupation and marital status. (3) That there were no statistically significant differences between the participants assessed in Time 1 (state of alarm) and those evaluated in Time 2 (“new normal”) in age, educational level, occupation and marital status. (4) That their questionnaire did not have missing values. (5) That the number of women and men in each phase was the same. Given that, in the global sample, there were more women than men and there were more participants during Time 2 than during Time 1, the sample of men in Time 1 that met the criteria was first selected. Their number, age, educational level, marital status and occupation were analyzed with the categories described in Table 1 and these values were set as the criteria for random selection of the other three study groups (Time 1 women and Time 2 women and men).

All procedures performed in the study were in accordance with the ethical standards of the institutional and/or national research committee and with the 1964 Helsinki declaration and its further amendments or comparable ethical standards and participants were also treated in accordance with APA research guidelines. All participants gave consent and had the possibility to cancel their participation at any time. The research was approved by the Ethics Commission for Research with Human Beings (CEIH) of the University of Pablo de Olavide of Seville (code 21/8 − 6).

## Measures

### Mental distress

Participants’ mental distress was assessed by using the Spanish version of the 12-item General Health Questionnaire (GHQ) (Goldberg et al., 1996). The GHQ-12 is a short screening instrument designed to detect current non-specific mental disturbance in primary care settings and in the general population (Goldberg et al., 1997). In this study items were scored according to the Likert method that assigns a weight to each score, from 0 to 3 (where 0 indicates no distress or reduced function), so the total scores ranged from 0 to 36 and higher scores indicated higher levels of mental distress. According to Lundin et al. (2017) the best threshold to discriminate mental distress cases from non-cases for the Likert scoring method of GHQ-12 was ≥ 14 (sensitivity = 85.5 and specificity = 83.2). For the present sample the Cronbach’s α reliability test of 12 items was 0.90 and McDonald’s ω reliability coefficient was 0.89.

### Positive and negative experience and affect balance

The Spanish version of the Scale of Positive and Negative Experience (SPANE, Diener et al., 2010) was used to assess participants’ positive and negative feelings and affect balance. The SPANE is a 12-item scale, with six items designed to assess positive experiences (SPANE-P) and six items devoted to negative experiences (SPANE-N) that, together, reflect all types of feelings and “assesses the full range of possible desirable and undesirable experiences” (Diener et al., 2010, p. 145). The positive and negative scales are scored separately and the two scores can be combined in the affect balance measure (SPANE-B) by subtracting the negative score from the positive score, assessing in this way the preponderance of positive affect over negative affect. The resultant SPANE-B score can vary from − 24 (unhappiest possible) to 24 (highest affect balance possible).

This scale has been validated in several countries, including Spain (Espejo et al., 2020) and has shown good psychometric properties. In our study, Cronbach’s α for the positive feelings had a value of 0.91 and 0.87 for negative feelings, the same coefficient values were obtained using McDonald´s ω reliability.

### Self-esteem

The Spanish version of the Rosenberg Self-Esteem Scale (Rosenberg, 1965), adapted by Martín-Albo et al. (2007), was used to assed self-esteem. This is a ten item scale that assesses global self-esteem. The scale contains items such as “On the whole, I am satisfied with myself” and “I take a positive attitude toward myself”. Participants were asked to rate each item on a four-point scale from 0 (*strongly agree*) to 3 (*strongly disagree*) and higher scores indicate higher levels of self-esteem. For the present sample, both the Cronbach’s α and McDonald’s ω reliability coefficients were 0.84.

### Social support

The Social Support Scale (Matud, 1998) is a self-report measure that assesses perceived availability of social support (Matud et al., 2003). It consists of 12 items which gather information about the possibility of access to other persons who can provide emotional and instrumental support. The emotional social support subscale consists of 7 items such as “Someone who listens when you need to talk about your feelings” and “Someone with whom you can totally be yourself”. The instrumental social support scale consists of 5 items such as “Someone who lends you money when you have economic problems” and “Someone who helps you when you have work problems”. Response options range from 0 (*never*) to 3 (*always*) with higher scores indicating higher levels of social support. The present sample produced adequate-to-excellent internal consistency both for emotional social support (Cronbach’s α = 0.90; McDonald´s ω = 0.91), and instrumental social support (Cronbach’s α = 0.88; McDonald’s ω = 0.87).

### Socio-demographic measures

The participants` age was treated as a continuous variable whilst their educational level was approached as an ordinal variable with seven levels. Scores were assigned from 1 (for basic education) to 7 (for 5-year university degree), so high scores indicated a greater educational level. Other socio-demographic measured comprised gender, marital status, number of children and occupation.

### Data analysis

Internal consistency was measured using Cronbach’s Alpha and McDonald´s Omega (ω). General descriptive statistics were computed to describe the demographic characteristics of the participants. Comparison between women and men for age and number of children were computed by using Student’s *t* tests and for education, marital status, occupation and GHQ caseness by using the Pearson’s Chi-square test. To find out if there were differences between women and men in each phase and between the two phases analyzing each gender separately, four 2 × 2 between-subject analysis of variance (ANOVA) were performed. Independent variables were gender (women and men) and time (state of alarm –Time 1- and “new normal” -Time 2) and dependent variables were mental distress in the first ANOVA, positive feelings in the second, negative feelings in the third, affect balance in the fourth, self-esteem in the fifth, emotional social support in the sixth, and instrumental social support in the seventh. To achieve the second study’ aim, hierarchical multiple regression analyses were conducted separately for women and men in the state of alarm phase and in the “new normal” phase. The criterion considered was the score in mental distress and the score in affect balance. In each regression analysis, age, educational level and marital status as a dummy variable with two levels: one including people married or living with a partner, which was coded wit 1, and another including single, separated, divorced or widowed people, which was coded 0 and considered the reference category, were included in step 1, and self-esteem, emotional social support and instrumental social support scores were included in the second step. Statistical analyses were carried out using the software IBM SPSS Statistics for Windows, version 21.0 (IBM Corp., Armonk, N.Y., USA).

Additionally, we performed a sensitivity analysis using G*Power (version 3.1.9.7) for the ANOVA models.

## Results

The results for the G*Power sensitivity analysis for the ANOVA models indicated that, with the size of the sample in our study, a power of 95%, and alpha of 0.05, the sensitivity (effect size *f*) is 0.14.

### Gender differences in mental distress, positive and negative experiences and affect balance

**Fig. 1:**
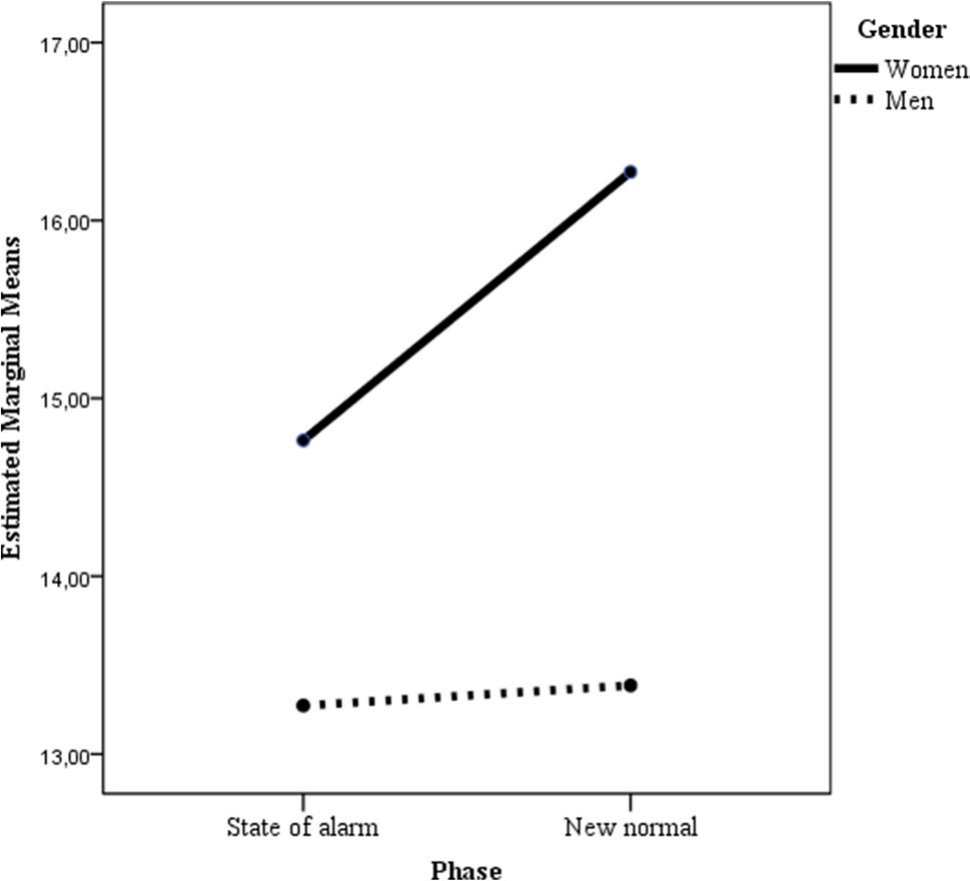
Two-way interaction of gender and time predicting psychological distress

Table 2 presents the results of two-factor ANOVAs (2 × 2) with participants’ gender (women vs. men) and Time (state of alarm vs. “new normal”) as between-subjects factors. When mental distress was considered as the dependent variable, the main effects of gender and time, as well as the gender x time interaction, were statistically significant. Bonferroni post hoc comparison to test for differences between groups showed statistically significant differences (*p* = .022) between women in Time 1 (state of alarm) and women in Time 2 (“new normal”), mean difference = -1.51; 95% CI [-2.88, -0.14]; women in Time 1 and men in Time 1 (*p* = .03), mean difference = 1.49; 95% CI [0.12, 2.86]; and women in Time 2 and men in Time 2 (*p* < .001), mean difference = 2.89; 95% CI [1.52, 4.26]. As can be seen in Fig. 1, women had more mental distress than men, both in the phase of state of alarm (Time 1) and in the “new normal” phase (Time 2), but while women in the latter phase had more mental distress that during state of alarm, in men their mental distress was very similar in both situations (mean difference = -0.11; 95% CI [-1.48, 1.26], *p* = 1.00).

**Table 2:** Means (*M*), standard deviations (*SD*), and two-way ANOVA statistics for the study variables

Variable	Women			Men	ANOVA			
	*M*	*SD*	*M*	*SD*	Effect	*F* ratio	*df*	η^2^
Mental distress								
Time 1	14.76	6.72	13.27	6.25	Gender	35.67***	1,1300	0.027
Time 2	16.27	6.94	13.39	6.54	Time	4.90*	1,1300	0.004
Interaction Gender x Time					G x T	3.63*	1,1300	0.003
Positive Feelings								
Time 1	20.95	4.03	20.55	3.97	Gender	0.02	1,1300	0.000
Time 2	20.62	4.39	20.95	3.91	Time	0.02	1,1300	0.000
Interaction Gender x Time					G x T	2.56	1,1300	0.002
Negative feelings								
Time 1	16.67	4.58	15.90	4.69	Gender	32.59***	1,1300	0.024
Time 2	17.18	4.60	15.08	4.28	Time	0.36	1,1300	0.000
Interaction gender x Time					G x T	7.01**	1,1300	0.005
Affect balance								
Time 1	4.28	7.76	4.66	7.69	Gender	10.99**	1,1300	0.008
Time 2	3.44	7.96	5.87	7.10	Time	0.18	1,1300	0.000
Interaction gender x Time					G x T	5.91*	1,1300	0.005
Self-esteem								
Time 1	20.31	5.03	20.85	4.65	Gender	8.22**	1,1300	0.006
Time 2	20.21	4.72	21.17	4.44	Time	0.17	1,1300	0.000
Interaction gender x Time					G x T	0.62	1,1300	0.000
Emotional social support								
Time 1	15.86	4.86	15.37	4.37	Gender	1.04	1,1299	0.001
Time 2	16.09	4.76	16.05	4.82	Time	3.07	1,1299	0.002
Interaction gender x Time					G x T	0.73	1,1299	0.001
Instrumental social support								
Time 1	10.15	4.19	8.59	3.75	Gender	20.71***	1,1300	0.016
Time 2	10.05	4.06	9.60	3.90	Time	4.13*	1,1300	0.003
Interaction gender x Time					G x T	6.35*	1,1300	0.005

N = 1304. ANOVA = Analysis of variance. Time 1 = state of alarm in Spain. Time 2 = “new normal” in Spain

* *p* < .05; ** *p* < .01; *** *p* < .001

The prevalence of mental distress in the state of alarm (Time 1) was 39.9% in men and 52.5% in women, differences in percentages that were statistically significant, χ2 (1, *N* = 652) = 10.37, *p* = .001. In the “new normal” phase (Time 2), the percentage of mental distress in men was 40.8% and in women 61.7%, differences in percentages that were also statistically significant, χ2 (1, *N* = 652) = 28.39, *p* < .001. The differences in the prevalence of mental distress between the alarm state and the “new normal” phases in men was not statistically significant, χ2 (1, *N* = 652) = 0.06, *p* = .81, but it was statistically significant in women, χ2 (1, *N* = 652) = 5.63, *p* = .02.

**Fig. 2:**
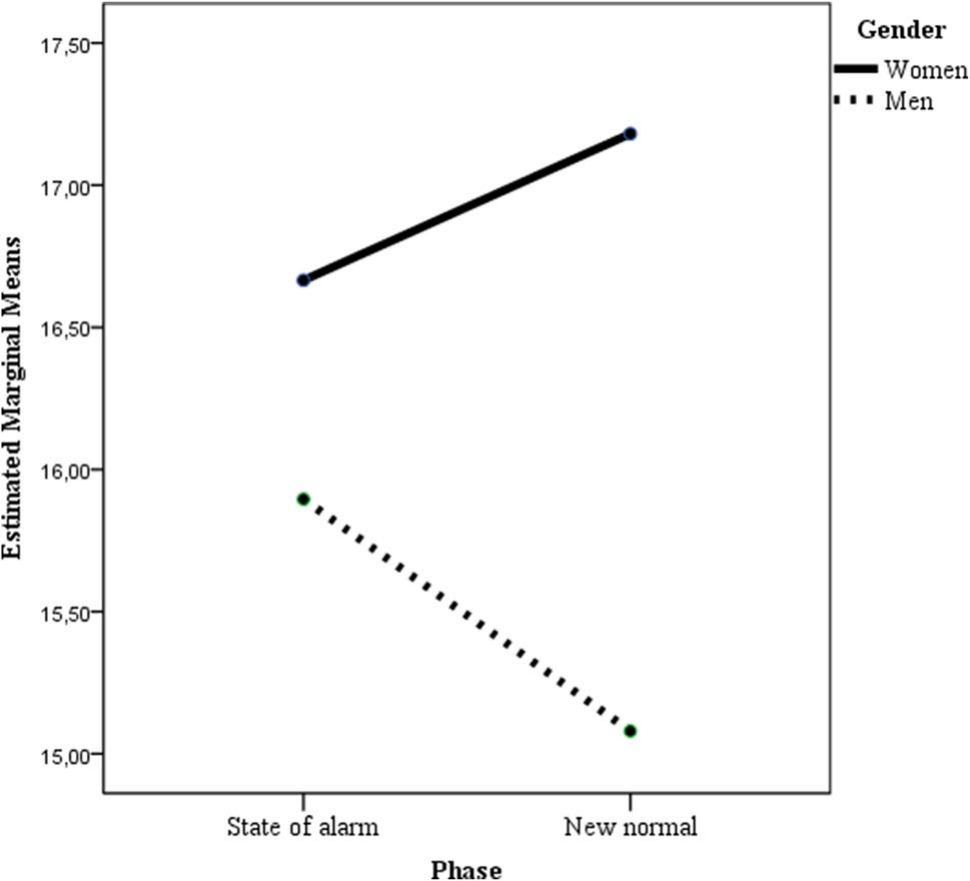
Two-way interaction of gender and time predicting negative feelings

In the ANOVA in which positive feelings was considered as the dependent variable, no effect was statistically significant, neither were the main effects of gender, nor that of time, nor was the interaction gender x time (see Table 2). When negative feelings score was considered as the dependent variable, the ANOVA results showed that only the main effects of gender and gender x time interaction were statistically significant (see Fig. 2). Bonferroni post hoc comparison to test for differences between groups showed only statistically significant differences (*p* < .01) between women in Time 2 and men in Time 2 (*p* < .001), mean difference = 2.10; 95% CI [1.16, 3.04].

**Fig. 3:**
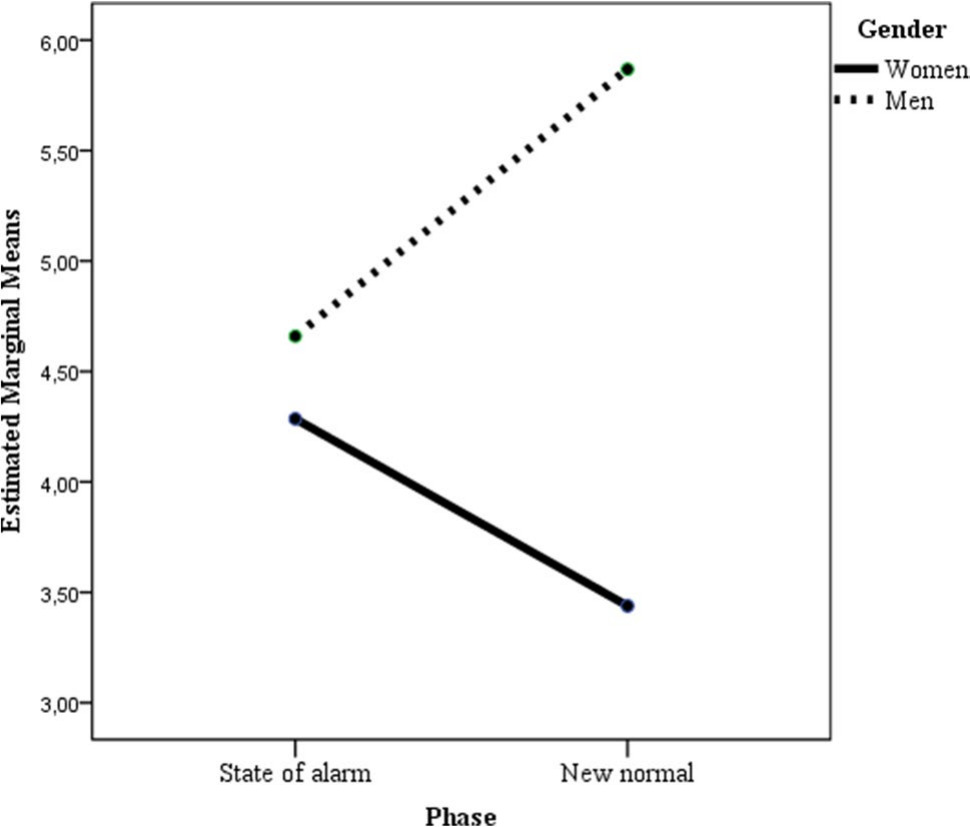
Two-way interaction of gender and time predicting affect balance

In the ANOVA in which affect balance was considered as the dependent variable, statistically significant main effects of gender and gender x time interaction were found (see Table 2; Fig. 3). Bonferroni post hoc comparison to test for differences between groups showed only statistically significant differences (*p* < .01) between women in Time 2 and men in Time 2, mean difference = -2.43; 95% CI [-4.00, -0.85].

**Fig. 4:**
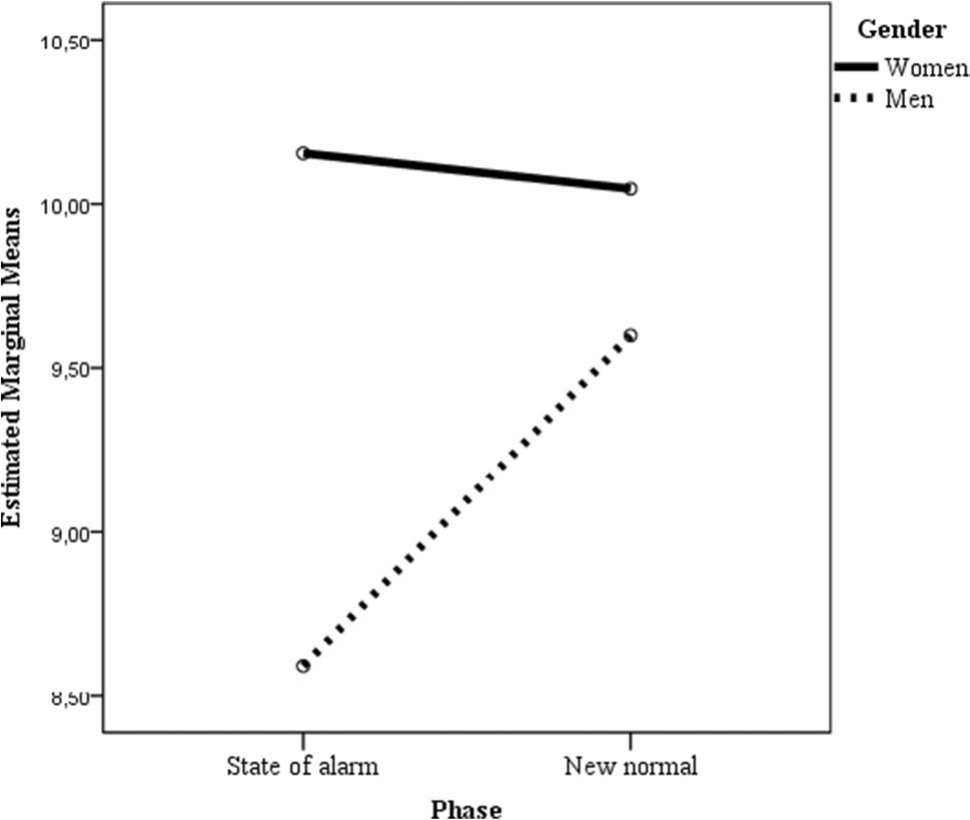
Two-way interaction of gender and time predicting instrumental social support

In the ANOVA in which self-esteem was considered as the dependent variable (see Table 2), only gender main effect was statistically significant. Women had less self-esteem (mean adjusted score = 20.26, 95% CI [19.90, 20.63] than men (mean adjusted score = 21.01, 95% CI [20.65, 21.37]. In the ANOVA in which emotional social support was considered as the dependent variable, no effect was statistically significant, neither were the main effects of gender, nor that of time, nor was the interaction gender x time. When instrumental social support was considered as the dependent variable both the gender and the time main effects were statistically significant, as well as the gender x time interaction. Bonferroni post hoc comparison to test for differences between groups showed statistically significant differences (*p* < .001) between women in Time 1 and men in Time 1, mean difference = 1.56; 95% CI [0.73, 2.38]; and men in Time 1 and men in Time 2 (*p* = .008), mean difference = -1.00; 95% CI [-1.83, -0.18] (see Table 2; Fig. 4).

### Protective role of age, educational level, marital status, self-esteem and social support against mental distress and as factors that increase the affect balance

Table 3 presents the hierarchical regression results with the mental distress as the dependent variable in state of alarm and in new normal phases in Spain for the women and men groups. In state of alarm, in both genders, results identified that *R* for regression was significantly different from zero only at step 2 in the male sample and at both steps in the female sample. Low self-esteem was a statistically significant predictor of higher mental distress in both genders. In the women group, another significant predictor of higher mental distress was lower emotional social support. Although being married or having a partner was statistically significant in association with less distress in step 1 in the sample of women, when self-esteem and social support were included in step 2, marital status was no longer statistically significant. In step 2, with all IVs in the equation, adjusted *R*^2^ was 0.36 for the women group and 0.30 for the men group, indicating that 36% of the variability in women’s mental distress and 30% of the men’s mental distress was predicted. At “new normal” phase results identified that *R* for regression were significantly different from zero only at the end of step 2. In the women group, the statistically significant variables were self-esteem and instrumental social support, and the percentage of variance explained was 41%. In the men group, the percentage of explained variance was 19% and those with more mental distress were those with less self-esteem and less emotional social support.

**Table 3:** Hierarchical regression result for mental distress for the women and men groups in state of alarm and in “new normal” phases in Spain

Variable		*B*	β		Semipartial correlations	*R* ^2^	Δ*R*^*2*^	*B*	β	Semipartial correlations	*R* ^2^	Δ*R*^*2*^
		State of alarm phase						New normal phase				
		Women										
Step 1						0.03	0.03*				0.02	0.02
Constant		16.39***						17.83***				
Age		− 0.02	− 0.05		− 0.05			− 0.05	− 0.10	− 0.09		
Education		0.12	0.02		0.02			0.34	0.04	0.04		
Married/partnered		-2.04	− 0.15**		− 0.15			-1.26	− 0.09	− 0.09		
Step 2						0.37	0.34***				0.42	0.39***
Constant		31.74***						34.86**				
Age		− 0.01	− 0.01		− 0.01			− 0.04	− 0.08	− 0.07		
Education		0.24	0.04		0.03			0.53	0.07	0.07		
Married/partnered		− 0.48	− 0.04		− 0.03			− 0.92	− 0.07	− 0.06		
Self-esteem		− 0.58***	− 0.43***		− 0.36			− 0.80***	− 0.54***	− 0.47		
Emotional Social support		− 0.32**	− 0.23**		− 0.12			− 0.16	0.11	− 0.06		
Instrumental Social support		− 0.05	− 0.03		− 0.02			− 0.47***	− 0.28***	− 0.16		
	Men											
Step 1						0.02	0.02				0.01	0.01
Constant		14.55***						11.22***				
Age		− 0.06*	− 0.13*		− 0.12			0.03	0.06	0.05		
Education		0.15	0.02		0.02			0.54	0.07	0.07		
Married/partnered		0.59	0.05		0.05			-1.03	− 0.08	− 0.07		
Step 2						0.32	0.30***				0.21	0.20***
Constant		28.59***						26.83***				
Age		− 0.03	− 0.06		− 0.06			− 0.01	− 0.03	− 0.01		
Education		0.57	0.09		0.08			0.57	0.08	0.07		
Married/partnered		0.93	0.08		0.07			-1.26	− 0.09	− 0.02		
Self-esteem		− 0.66***	− 0.50***		− 0.44			− 0.57***	− 0.38***	− 0.35		
Emotional Social support		− 0.21	− 0.15		− 0.09			− 0.23*	− 0.17*	− 0.10		
Instrumental Social support		0.03	0.02		0.01			0.08	0.05	0.03		

* *p* < .05; **p < .01; *** *p* < .001

Table 4 presents the hierarchical regression results with the affect balance as the dependent variable in the state of alarm and in “new normal” phases for the women and men groups. In state of alarm phase, for both genders *R* was significantly different from zero at the end of each step. Beta values in step 2, with all IVs in the equation, probed that self-esteem was the variable most associated with women’s and men’s affect balance. In the women group, emotional social support and education were also statistically significant predictors of affect balance, whilst in the men group age was another significant predictor. The adjusted *R*^2^ value for women and men were 0.38 and 0.37, respectively, indicating that over a third of the variability in the affect balance of women and men was predicted. At the “new normal” phase *R* for regression were significantly different from zero only at the end of step 2. The women with more affect balance were those with more self-esteem and more emotional social support. The adjusted *R*^2^ of 0.47 indicated that almost half of the variability in women’s affect balance was predicted. In the men group, the percentage of explained variance was 30% and those with more affect balance were men with more self-esteem and emotional social support.

**Table 4:** Hierarchical regression result for affect balance for the women and men groups in state of alarm and in “new normal” phases in Spain

Variable		*B*	β	Semipartial correlations	*R* ^2^	Δ*R*^*2*^	*B*	β	Semipartial correlations	*R* ^2^	Δ*R*^*2*^
		State of alarm phase						New normal phase			
	Women										
Step 1					0.04	0.04**				0.00	0.00
Constant		3.69					3.71				
Age		0.04	0.07	0.07			0.01	0.01	0.01		
Education		− 0.70	− 0.09	− 0.08			− 0.26	− 0.03	− 0.03		
Married/partnered		-2.32	0.15**	0.15			0.63	0.04	0.04		
Step 2					0.39	0.35***				0.47	0.47***
Constant		-13.98***					-20.02***				
Age		0.01	0.02	0.02			0.01	0.01	0.01		
Education		− 0.82*	− 0.10*	− 0.10			− 0.40	− 0.05	− 0.04		
Married/partnered		0.49	0.03	0.03			− 0.22	− 0.01	− 0.02		
Self-esteem		0.75***	0.49***	0.41			0.92***	0.55***	0.48		
Emotional Social support		0.29*	0.18*	0.09			0.25*	0.15*	0.08		
Instrumental Social support		0.04	0.02	0.01			0.21	0.11	0.06		
	Men										
Step 1					0.03	0.03*				0.02	0.02
Constant		1.92					7.18***				
Age		0.07*	0.13*	0.13			-03	− 0.05	− 0.05		
Education		− 0.17	− 0.02	− 0.02			− 0.46	− 0.06	− 0.06		
Married/partnered		1.05	0.07	0.07			2.11*	0.15*	0.14		
Step 2					0.38	0.35***				0.32	0.30***
Constant		-16.94***					-13.35***				
Age		0.04	0.08	0.08			0.03	0.07	0.06		
Education		− 0.69	− 0.08	− 0.08			− 0.57	− 0.07	− 0.07		
Married/partnered		0.54	0.04	0.03			0.99	0.07	0.06		
Self-esteem		0.80***	0.49***	0.44			0.66***	0.41***	0.37		
Emotional Social support		0.20	0.12	0.08			0.22*	0.15*	0.09		
Instrumental Social support		0.23	0.11	0.08			0.18	0.10	0.06		

*p < .05. **p < .01; *** *p* < .001

## Discussion

This study investigated gender differences in mental distress and in the affective component of subjective well-being (positive and negative feelings and affect balance) in the Spanish general population in two phases of the first wave of the COVID-19 pandemic in Spain: at the end of the state of alarm, from June 1st to June 20th, 2020, a phase in which, though some of the national quarantine measures had been relaxed, there were important restrictions, and at the end of the phase of “new normal”, from October 1st to October 20th, 2020, a phase where all activities were allowed, though maintaining a social distance of 1.5 m, taking hygienic measures and the compulsory use of facemask. Results show greater mental distress in women than in men, but the magnitude of such differences varies according to the phase studied. Men’s mental distress was very similar in both phases, 39.9% of the men experienced significant mental distress (GHQ-28´s score ≥ 14) at the end of the state of alarm and 40.8% in the “new normal” phase whereas the percentages of women who experienced significant mental distress were 52.5% at the end of the state of alarm and 61.7% in the “new normal” phase. These findings are in line with those found in other countries during the first wave of COVID-19 where greater psychological distress has been found in women compared to men (Pierce et al., 2020; Qiu et al., 2020; Rens et al., 2021). Up to now, it is not possible to draw conclusions regarding whether the greater distress of women compared to men is specific to the COVID-19 pandemic, since studies carried out before the pandemic have also found that women have more distress than men (Drapeau et al., 2010; Matud et al., 2015). In any case, the presence of greater distress in women, but not in men, in the “new normal” phase with respect to the state of alarm indicates that the COVID-19 pandemic poses a greater risk of psychological distress for women than for men.

The reason why women suffered greater mental distress in the “new normal” phase, when for example the care responsibilities were less than in the state of alarm phase, is not known. However, it is not clear that women’s care responsibilities decreased in this phase compared to the previous one and, on top of that, the accumulated effect of the time spent in lockdown, plus the higher risk of some members of the household becoming infected in this “new normality” phase, especially children and/or older adults, and the lack of vaccine protection at that point in time may all account for the higher burden for women.

The results of the analysis of gender differences in the affective component of subjective well-being show that, although positive feelings were very similar in women and men and in both phases of the first wave of the pandemic, women experienced more negative feelings than men, although the differences were only statistically significant in the “new normal” phase. This seems to be a consequence of the fact that negative feelings increased slightly in women in the “new normal” phase with respect to the state of alarm but decreased in men, although the differences between the two phases were not statistically significant neither in women nor in men. In addition, in the new normal phase, women had less affect balance than men. All this indicates that the COVID-19 pandemic poses a greater risk to the mental health and well-being of women than men in Spain. The results of this study increase the evidence that the COVID-19 pandemic poses a greater risk to the mental health and well-being of women, an issue that has already been cited by other authors (Connor et al., 2020; Kolakowsky-Hayner et al., 2021). Among the factors that have been proposed as explanatory of this greater risk for women, is the fact that parenting can be more stressing during a pandemic and that the pandemic has meant a greater need for care for sick and older adults, women being more involved than men in domestic chores and child and sick care (United Nations, 2020). In addition, women constitute the majority of the healthcare workforce, a sector that is at greater risk of illness and work overload due to the pandemic; on the other hand, the pandemic has restricted the access to the health and social services systems, limiting preventive and reproductive healthcare, as well as the attention to victims of intimate partner and gender-based violence, violence that has increased during the pandemic (Castellanos-Torres et al., 2020; Chang, 2020; Connor et al., 2020). Finally, it has also been suggested that women have been more affected than men due to situations of vulnerability derived from measures to contain the spread of the pandemic, such as the closure of educational centers, the restriction in access to services, especially health, and the partial closure of some economic activities whose workers are mostly women, such as hospitality or tourism (Castellanos-Torres et al., 2020).

The second aim or our study was to determine the protective role of age, educational level, marital status, self-esteem and social support against mental distress and as factors that increase the affect balance of women and men in the two phases of the first wave of COVID-19 pandemic in Spain: in the state of alarm and in the “new normal”. The regression analyses show that, in both phases and in both women and men, self-esteem was the best predictor of both mental distress and affect balance, with less distress and greater affect balance in women and men with higher self-esteem. Emotional social support also appeared as a protective factor against mental distress and as a factor that increased affect balance, although its relevance was much less than that of self-esteem. In addition, in women in the new normal phase, the perceived social support that predicted less distress was instrumental support, not emotional support, as was the case during the state of alarm. This could indicate that the COVID-19 pandemic implies a greater burden for women as the pandemic progresses and they have to face the new reality of living with it, which is why the perception of having instrumental social support is what acts as a protective factor against mental distress. On the contrary, in men, the perceived social support that seems to act as a protector against distress in this phase is emotional social support. In any case, the results of this study show that perceived social support is a protective factor on mental health during the COVID-19 pandemic, results that match those found in other countries (Grey et al., 2020; Li et al., 2021; Oryan et al., 2021).

In the present study, age was only a significant predictor of affect balance in men in the state of alarm phase, with greater affect balance in older men but Beta weight was low (0.13). The educational level was only a statistically significant predictor of the affect balance of women in the alarm state, with a higher balance in women with a lower level of education, although the Beta weight was only − 0.10. Although studies conducted in other countries have found that young people have a higher risk of mental symptoms (Gibson et al., 2021; Huang & Zhao, 2020; Oryan et al., 2021; Solomou & Constantinidou, 2020), such a result has not been confirmed in the present study. When predicting mental distress and affect balance, marital status was only statistically significant in the group of women and in the state of alarm phase in step 1, when being married or having a partner was associated with less mental distress and greater affect balance. However, this association was statistically significant when only sociodemographic variables were included in the regression equation, but it ceased to be statistically significant in step 2, when self-esteem and social support were included. This seems to indicate that the protective effects of being married or having a partner are limited to women and their effect may be due to other factors such as self-esteem and social support. Although some studies conducted during the COVID-19 pandemic have found that marriage is a protective factor against mental distress (Fernández et al., 2020; Hearne, 2021), there is evidence that such protection may depend on other variables. Thus, Hearne (2021) found that, although marriage was associated with less distress, married black women reported greater mental distress than other groups.

### Limitations and future research directions

This study has several limitations. The first is that it is a cross-sectional study, therefore, it does not provide information on how much participant´s mental distress and affect balance are increased by the COVID-19 pandemic. In addition, all measurements were obtained through self-reports, the sample was voluntary, and the link was sent to many people who in turn re-send it to other people. To protect anonymity, the details of the people to whom the link was sent were not recorded, nor were the number of links sent that were not acted upon or the reasons for rejection. Another limitation is that data collection was not completely done by random sampling and men and woman with university degrees are overrepresented in the sample. Furthermore, only the two phases of the first wave of the pandemic in Spain have been studied and the pandemic has gone through more waves in which there may be changes.

Future research could go deeper into the processes underlying the gendered impact of COVID-19. An analysis of differential exposition/perception of the pandemic stressors could be a suggested line. For example, what are the COVID-19 stressors that may be amplifying the impact on women through time? Are they mainly related to financial/job issues, to interpersonal conditions, to caregiving or parenting duties? Is the so-called “triple burden” for women rising up as the pandemic evolves? In general, the analysis of what is changing differentially for women and men in the COVID-19 era will be a worthy line of research. In a similar vein, future research should explore if/how COVID-19 differentially depletes the psychological resources of women and men. Previous research has already shown that women and men deal differently with adverse situations (Matud, 2004; Tamres et al., 2002). The study of the coping strategies displayed by women and men through COVID-19 phases may be another meaningful way of research, with substantial implications. Additionally, the inclusion of other variables, such as coping strategies and the additional stressors experienced in the new normal phase, could improve the prediction of psychological distress in men, since the variables used in our study only explained 19% of the variance.

Future studies should also consider the different waves of the pandemic, within the context of each country. Likewise, longitudinal studies, with measures that complete the self-reports could shed more light on the results found. In any case, matching the samples between men and women, as well as in variables such as age, number of children, education level and occupation, is important when it comes to generalizing and making the results comparable.

## Conclusions

The COVID-19 pandemic is a major threat to the health and well-being of people around the world that has impacted not only physical health, but has also significantly altered people’s lives, also affecting work conditions and the economy. But the pandemic does not seem to have affected women and men equally and, as a consequence, determining the degree to which the pandemic is affecting women and men differently is necessary to understand the effect of the pandemic on the mental health of individuals and communities and for designing effective interventions (Whenham et al., 2020). In this context, the results of the present study indicate that women in Spain not only are at greater risk of mental distress than men during the COVID-19 pandemic, but that this risk increases as the pandemic progresses. In addition, our study also shows that the COVID 19 pandemic poses a greater threat to the emotional component of women’s subjective well-being compared to men.

Finally, the results of this study indicate the relevance of high self-esteem and, to a lesser extent, of social support, as protective factors against mental distress and as promoters of the affect balance of women and men during the COVID-19 pandemic. Altogether, the results of this study are relevant for the design of policies and programs aimed at improving the health of the population and preventing gender differences in health from continuing to widen during the COVID-19 pandemic.

## Data Availability

The datasets generated and analyzed during the current study are available from the corresponding author on reasonable request.

## Declarations

This study was approved by the appropriate ethics committee and was conducted in accordance with the ethical standards laid down in the 1964 Declaration of Helsinki and its later amendments.
